# Efficient multiscale calculation results for microchannel mass transfer

**DOI:** 10.1038/s41598-021-89447-2

**Published:** 2021-05-11

**Authors:** Yongbin Zhang

**Affiliations:** College of Mechanical Engineering, Changzhou University, Changzhou, Jiangsu Province, 213164 China

**Keywords:** Fluid dynamics, Computational nanotechnology

## Abstract

When the channel height is reduced to a small value such as on the scales of 10 nm or 100 nm, the physical adsorbed layers on the channel walls will participate in the flow, although intermediate between them is a continuum fluid flow. The multiscale simulation results are presented for this multiscale mass transfer in a narrow slit pore based on the derived flow equations. The results are respectively compared with those calculated from conventional continuum flow theory and from the theory based on the solid layer assumption, when the fluid-wall interaction is respectively weak, medium and strong. The total mass flow rate of the two adsorbed layers is also compared with the mass flow rate of the intermediate continuum fluid. The obtained results show the importance of the incorporation of the adsorbed layer flow by the multiscale scheme when calculating the transferred mass in a microchannel.

## Introduction

Mass transfer in a macro channel can be calculated from conventional hydrodynamic flow theories, which were developed for continuum flows^1–3^. For a nanochannel flow, molecular dynamics simulation was often adopted to calculate the flow velocity profile^4–8^. For a pressure driven nanochannel flow, it was found that the volume flow rate through the channel calculated from molecular dynamics simulation is considerably smaller than that calculated from conventional hydrodynamic theory when the fluid-wall interaction is quite strong^9^; the reduced flow rate in the nanochannel was ascribed to the formation of the strongly solidified layer on the wall surface, which makes the channel narrower^9^. Besides full atomistic molecular dynamics simulation, there are also other methods proposed for simulating a nanochannel flow such as the dissipative particle dynamics method^10^, the quasi-continuum model^11^, the modified Navier–Stokes model^12^, the lattice Boltzman method^13^, the multiscale hybrid model^14^ and the flow factor approach model^15^.

There is the channel flow which intervenes between macrochannel flow and nanochannel flow. The channel height in this channel flow may be on the scales of 10 nm or 100 nm. In this flow, the thickness of the adsorbed layer on the wall is comparable with the film thickness of the intermediate continuum fluid, and the effect of the adsorbed layer should be considerable. Currently lacking is an efficient computational method for such a channel flow. Full atomistic molecular dynamics simulation seems difficult to handle this flow because of the over big channel size. Conventional hydrodynamic flow theory may also not be sufficiently accurate for describing this flow because of the influence of the adsorbed layer, which is actually not in a continuum flow. There have been the multiscale simulations which treated the adsorbed layer flow with full atomistic molecular dynamics simulation and treated the intermediate continuum fluid flow with the continuum model^14,16–18^. However, these multiscale approaches are currently only able to compute a tiny zone because of the limitation of the computer storage and the computational time, and they still can not solve an engineering problem because of the over large computation.

The present paper shows the simulation results by a novel multiscale analysis for the above mentioned multiscale channel flow. In the present analysis, the flow factor approach model for nanoscale flow is implemented for the adsorbed layer, while the continuum model is implemented for the intermediate continuum fluid. By this way, three closed-form explicit flow equations are respectively given for the flows of the two adsorbed layers and the flow of the continuum fluid. For a pressure driven channel flow, these flow equations were further simplified, and the solutions were then obtained by direct calculation to show the effect of the adsorbed layer on the mass flow rate through the channel for different fluid-wall interactions. The efficiency of the present multiscale scheme is thus directly evident. Important conclusions have been drawn concerning the effect of the adsorbed layer in multiscale mass transfer.

## Studied channel flow

**Figure 1:**
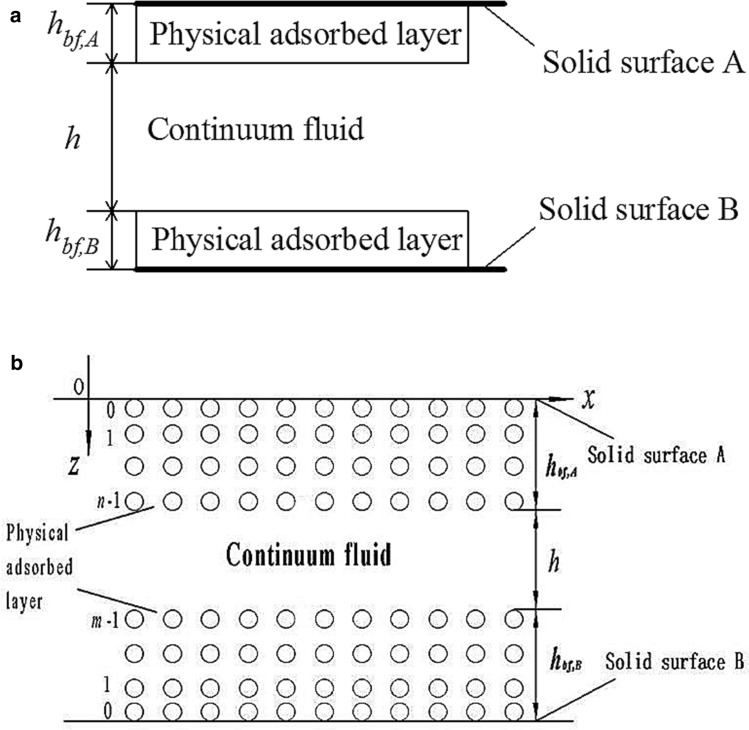
The studied flow in a narrow slit pore^22^ (with permission from the publisher). The pressure-driven fluid flow in a narrow slit pore where the thicknesses ($h_{bf,A}$ and $h_{bf,B}$) of the physical adsorbed layers on the walls are comparable with the film thickness ($h$) of the continuum fluid which intervenes between the two adsorbed layers A and B.

Figure 1a shows the studied pressure-driven fluid flow in a narrow slit pore where the thicknesses ($h_{bf,A}$ and $h_{bf,B}$) of the physical adsorbed layers on the walls are comparable with the film thickness ($h$) of the continuum fluid which intervenes between the two adsorbed layers A and B. In this case, the effect of the adsorbed layer on the channel flow should be considered. When the flow rate of the adsorbed layer is far smaller than that of the continuum fluid, the adsorbed layer can be treated as a solid layer; otherwise, there should be a method to simulate the adsorbed layer flow. A conventional multiscale approach may calculate both the adsorbed layer flows by full atomistic molecular dynamics simulation and calculate the continuum fluid flow by the continuum fluid model^14,16–18^. As commented above, such an approach usually requires an unaffordable computational time and storage for an engineering flow. The reason for this difficulty is that when carrying out a molecular dynamics simulation for the adhering layer flows, there are a huge amount of molecules both in the adhering layer zones and in the solid wall zones involved because of the macroscopic length in the flow direction; accounting for the pairwise interactions between all the fluid molecules and between all the fluid and solid wall molecules by molecular dynamics simulation is really a massive computational work as having been well known. The conventional multiscale approach with molecular dynamics simulation is actually the numerical iteration calculation with discretized grids in the flow zones. It undoubtedly takes unendurable computational costs for tremendous discretized volumes in an engineering multiscale flow. This problem has prevented us from further understanding the multiscale flow even in a relatively long channel. For trying to overcome it, other hybrid approaches such as atomistic and coarse-grained hybrid methods have also been proposed^19,20^. However, they are also not satisfying for solving a long channel flow because of the low efficiency. For an engineering multiscale flow such as shown in Fig. 1a, the classical multiscale approaches with molecular dynamics simulation involved are actually unable to give the final solution because of the current computational capacity limited.

With an essentially different way, the present method analytically derives the flow equations respectively for the two adhering layer flows and the intermediate continuum fluid flow all of which are closed-form and explicit. In the present multiscale calculation, no numerical iterations for massive discretized molecules (as occurring in molecular dynamics simulation) are present, the final solution is given by a direct calculation from the analytical equations which are explicit, and the efficiency are thus radically improved.

The present study calculates the flow velocity and flow rate of the adsorbed layer by the flow factor approach model^15^ and calculates the intermediate continuum fluid flow by the continuum fluid model. In doing this, both the adsorbed layers are treated as equivalent ordered molecules normal to the wall surfaces and intervening between them is the continuum fluid, as shown in Fig. 1b. When $h$ is vanishing and the channel flow becomes the nanoscale non-continuum flow, the flow factor approach model has been well verified as compared with the molecular dynamics simulation results^15^. The treatment of the adsorbed layer by the flow factor approach model in the present study as shown in Fig. 1b should also be reliable. As a fundamental work, here it is assumed that neither interfacial slippage occurs on the adsorbed layer-fluid interface or on the adsorbed layer-wall surface interface.

## Analysis

### For the adsorbed layer treated as a solid layer

For comparison, the analytical results are first derived in this section when the adsorbed layer is treated as a solid layer. This treatment for the physical adsorbed layer has been done in the earlier work^21^.

Conventional hydrodynamic flow theory ignores the physical adsorbed layer on the wall surface. For the pressure driven flow shown in Fig. 1a, in the case of no interfacial slippage, the theory gives the following mass flow rate per unit channel width through the channel:

1 $$ q_{m,conv} = - \frac{{\rho (h + h_{bf,A} + h_{bf,B} )^{3} }}{12\eta }\frac{\partial p}{{\partial x}} $$

where $p$ is the pressure, $\rho$ and $\eta$ are respectively the fluid bulk density and bulk viscosity, and $x$ is the coordinate in the flow direction.

When the physical adsorbed layers on both the wall surfaces are considered as solid layers, the mass flow rate per unit channel width through the channel is calculated as:

2 $$ q_{m,s} = - \frac{{\rho h^{3} }}{12\eta }\frac{\partial p}{{\partial x}} $$

Define $\lambda_{bf} = h_{bf,A} /h$. If $h_{bf,A} = h_{bf,B}$, it is obtained that:

3 $$ r_{q,s} = \frac{{q_{m,s} }}{{q_{m,conv} }} = \frac{1}{{(1 + 2\lambda_{bf} )^{3} }} $$

### For the adsorbed layer treated as a flowing layer

In themselves, both the physical adsorbed layers A and B in Fig. 1a should be treated as flowing layers as they can somewhat flow by the pressure driving, depending on the layer solidification. The flow factor approach model^15^ treats both the adsorbed layers as the equivalent ordered molecules as shown in Fig. 1b.

The two fundamental equilibrium equations for the ordered molecules on the wall surfaces are respectively^15^:

4 $$ \frac{\partial p}{{\partial x}} = \frac{{\delta \tau_{i} }}{D} $$

and

5 $$ \tau_{i - 1} = \eta_{line,i - 1} \frac{{\delta u_{i} }}{{\Delta_{i - 1} }} $$

where *D* is the fluid molecule diameter, $\delta \tau_{i}$ is the shear stress difference between the *i*th and (*i*-1)th fluid molecules, $\eta_{line,i - 1}$, $\Delta_{i - 1}$ and $\delta u_{i}$ are respectively the local viscosity, the separation and the velocity difference between the *i*th and (*i*-1)th fluid molecules across the layer thickness, and $i$ is the order number of the molecule across the layer thickness as shown in Fig. 1b.

Based on Eqs. (4) and (5), three flow equations have been derived respectively for the two adsorbed layers A and B and the intermediate continuum fluid^22^. If $h_{bf,A} = h_{bf,B}$ and the physical adsorbed layer A is identical with the physical adsorbed layer B, the volume flow rates per unit channel width of both the adsorbed layers are equated as^22^:

6 $$ q_{v,bf} = [\frac{{F_{1} h_{bf,A}^{3} }}{{12\eta_{bf}^{eff} }}{ - }\frac{{h_{bf,A}^{3} }}{{2\eta_{bf}^{eff} }}(1 + \frac{1}{{2\lambda_{bf} }} - \frac{{q_{0} - q_{0}^{n} }}{{q_{0}^{n - 1} - q_{0}^{n} }}\frac{{\Delta_{n - 2} }}{{h_{bf,A} }})\frac{\varepsilon }{{1 + \frac{\Delta x}{D}}}]\frac{\partial p}{{\partial x}} $$

where $$\eta_{bf}^{eff}$$ is the effective viscosity of the physical adsorbed layer and $$\eta_{bf}^{eff} = Dh_{bf,A} /[(n - 1)(D + \Delta_{x} )(\Delta_{l} /\eta_{line,l} )_{avr,n - 1} ]$$^15^, $$\Delta x$$ is the separation between the neighboring fluid molecules in the *x* coordinate direction in the adsorbed layer, $$q_{0} = \Delta_{j + 1} /\Delta_{j}$$ and $$q_{0}$$ is constant^15^, $$F_{1} = \eta_{bf}^{eff} (12D^{2} \psi + 6D\varphi )/h_{bf,A}^{3}$$^15^, and $$n$$ is the number of the fluid molecules across the layer thickness. Here,$$I = \sum\limits_{i = 1}^{n - 1} {i(\Delta_{l} /\eta_{line,l} )_{avr,i} }$$, $$II = \sum\limits_{i = 0}^{n - 2} {[i(\Delta_{l} /\eta_{line,l} )_{avr,i} + (i + 1)(\Delta_{l} /\eta_{line,l} )_{avr,i + 1} ]\Delta_{i} }$$, $$\psi = \sum\limits_{i = 1}^{n - 1} {i(l\Delta_{l - 1} /\eta_{line,l - 1} )_{avr,i} }$$, $$\varphi = \sum\limits_{i = 0}^{n - 2} {[i(l\Delta_{l - 1} /\eta_{line,l - 1} )_{avr,i} + (i + 1)(l\Delta_{l - 1} /\eta_{line,l - 1} )_{avr,i + 1} ]\Delta_{i} },$$$$i(\Delta_{l} /\eta_{line,l} )_{avr,i} = \sum\limits_{j = 1}^{i} {\Delta_{j - 1} /\eta_{line,j - 1} },$$ and $$i(l\Delta_{l - 1} /\eta_{line,l - 1} )_{avr,i} = \sum\limits_{j = 1}^{i} {j\Delta_{j - 1} /\eta_{line,j - 1} }$$^15^.

In this case, the volume flow rate per unit channel width of the continuum fluid is^22^:

7 $$ q_{v,hf} = \{ - \frac{{h^{3} }}{12\eta } + \frac{{h^{3} }}{{\eta_{bf}^{eff} }}[\frac{{F_{2} \lambda_{bf}^{2} }}{6} - \frac{{\lambda_{bf} }}{{1 + \frac{\Delta x}{D}}}(\frac{1}{2} + \lambda_{bf} - \frac{{q_{0} - q_{0}^{n} }}{{q_{0}^{n - 1} - q_{0}^{n} }}\frac{{\Delta_{n - 2} }}{h})]\} \frac{\partial p}{{\partial x}} $$

where $F_{2} = 6\eta_{bf}^{eff} D(n - 1)(l\Delta_{l - 1} /\eta_{line,l - 1} )_{avr,n - 1} /h_{bf,A}^{2}$^15^. Equation (7) is radically different from any conventional continuum flow equation because of the incorporation of the coupled effect term. It is also obvious that conventional hydrodynamic flow theory is essentially inadequate for calculating the intermediate continuum fluid flow in the present mass transfer.

The total mass flow rate per unit channel width through the channel is thus:

8 $$ q_{m,total} = 2\rho_{bf}^{eff} q_{v,bf} + \rho q_{v,hf} $$

where $\rho_{bf}^{eff}$ is the average density of the physical adsorbed layer across the layer thickness.

It is defined that $r_{q} = q_{m,total} /q_{m,conv}$. For showing the proportion of the total mass flow rate of the two adsorbed layers, it is introduced that $r_{b/h} = 2\rho_{bf}^{eff} q_{v,bf} /(\rho q_{v,hf} )$.

### Parameter formulation

In the calculation, it was taken that $\Delta x/D = \Delta_{n - 2} /D = 0.15$,$\Delta_{j + 1} /\Delta_{j} = q_{0} ( > 1)$, and $\eta_{line,i} /\eta_{line,i + 1} = q_{0}^{m}$, where $q_{0}$ and $m$ are respectively positive constants^15^.

It was formulated that^23^:

9 $$ Cq(H_{bf} ) = \frac{{\rho_{bf}^{eff} (H_{bf} )}}{\rho } = m_{0} + m_{1} H_{bf} + m_{2} H_{bf}^{2} + m_{3} H_{bf}^{3} $$

where $H_{bf} = h_{bf,A} /h_{cr,bf}$, $h_{cr,bf}$ is a critical thickness, and $m_{0}$, $m_{1}$, $m_{2}$ and $m_{3}$ are respectively constant.

It was also formulated that^23^:

10 $$ Cy(H_{bf} ) = \frac{{\eta_{bf}^{eff} (H_{bf} )}}{\eta } = a_{0} + \frac{{a_{1} }}{{H_{bf} }} + \frac{{a_{2} }}{{H_{bf}^{2} }} $$

where $a_{0}$, $a_{1}$ and $a_{2}$ are respectively constant.

The regressed equations for calculating $\varepsilon$, $F_{1}$ and $F_{2}$ are respectively^22^:

11 $$ \varepsilon = (4.56E - 06)(\Delta_{n - 2} /D + 31.419)(n + 133.8)(q_{0} + 0.188)(m + 41.62) $$

12 $$ F_{1} = 0.18(\Delta_{n - 2} /D - 1.905)(\ln n - 7.897) $$

and

13 $$ F_{2} = ( - 3.707E - 04)(\Delta_{n - 2} /D - 1.99)(n + 64)(q_{0} + 0.19)(m + 42.43) $$

It has been found that Eqs. (11)–(13) have satisfactory calculation accuracies for practical cases^22^.

In the calculation, the weak, medium and strong fluid-wall interactions respectively have the following characteristic parameter values:

Weak interaction: *m* = 0.5, *n* = 3, $q_{0} = 1.03$, $h_{cr,bf}$ = 7 nm.

Medium interaction: *m* = 1.0, *n* = 5, $q_{0} = 1.1$, $h_{cr,bf}$ = 20 nm.

Strong interaction: *m* = 1.5, *n* = 8, $q_{0} = 1.2$, $h_{cr,bf}$ = 40 nm.

The other parameter values are shown in Tables 1 and 2.

**Table 1:** Fluid viscosity data for different fluid-wall interactions^21^.

Parameter interaction	a_0_	a_1_	a_2_
Strong	1.8335	− 1.4252	0.5917
Medium	1.0822	− 0.1758	0.0936
Weak	0.9507	0.0492	1.6447E− 4

**Table 2:** Fluid density data for different fluid-wall interactions^21^.

Parameter interaction	m_0_	m_1_	m_2_	m_3_
Strong	1.43	− 1.723	2.641	− 1.347
Medium	1.30	− 1.065	1.336	− 0.571
Weak	1.116	− 0.328	0.253	− 0.041

### Calculation results

**Figure 2:**
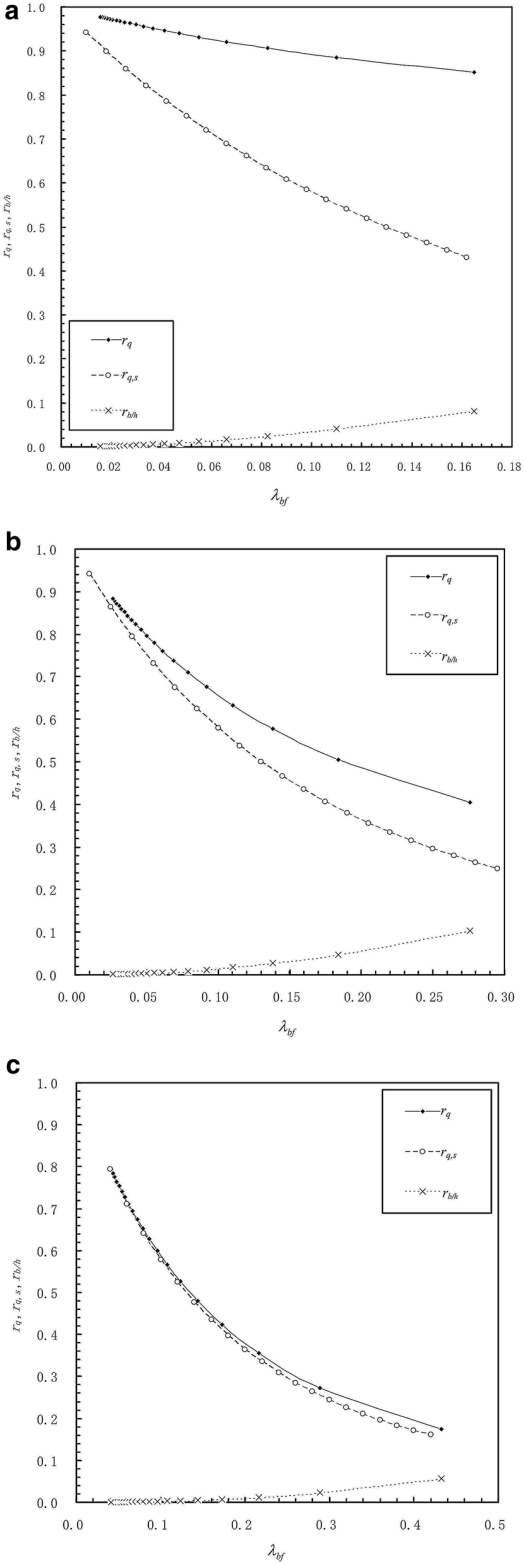
(**a**) The calculated values of $r_{q}$, $r_{q,s}$ and $r_{b/h}$ for the weak fluid-wall interaction when $m = 0.5$, $n = 3$, $q_{0} = 1.03$, and $\Delta_{n - 2} /D = 0.15$. When the fluid-wall interaction is weak, the present calculation is close to the conventional continuum flow calculation, and this indicates that the physical adsorbed layer is not so thick and it can flow well; treating the physical adsorbed layer as a solid layer will result in an erroneous calculation of the flow rate through the channel and it will severely underestimate the flow rate for $\lambda_{bf} \ge 0.03$; only for a very small $\lambda_{bf}$ (below 0.01) i.e. for a sufficiently large $h$, the physical adsorbed layer can be treated as a solid layer. (**b**) The calculated values of $r_{q}$, $r_{q,s}$ and $r_{b/h}$ for the medium fluid-wall interaction when $m = 1.0$, $n = 5$, $q_{0} = 1.1$, and $\Delta_{n - 2} /D = 0.15$. For the medium fluid-wall interaction, the effect of the adsorbed layer is significantly stronger than for the weak fluid-wall interaction; conventional hydrodynamic flow theory much overestimates the flow rate through the channel for $\lambda_{bf} \ge 0.05$ owing to ignoring the adsorbed layer effect, which significantly reduces the flow rate as shown by the values of $r_{q}$(< 0.8). (**c**) The calculated values of $r_{q}$, $r_{q,s}$ and $r_{b/h}$ for the strong fluid-wall interaction when $m = 1.5$, $n = 8$, $q_{0} = 1.2$, and $\Delta_{n - 2} /D = 0.15$. When the fluid-wall interaction is strong, for the same value of the continuum fluid film thickness $h$, the values of $r_{q}$ are significantly lower than those for the weak and medium fluid-wall interactions; this shows the strong effect of the adsorbed layer, which greatly reduces the flow rate through the channel; the curves for $r_{q}$ and $r_{q,s}$ are quite close for the plotted $\lambda_{bf}$ values.

Figure 2a shows the calculated values of $r_{q}$, $r_{q,s}$ and $r_{b/h}$ for varying $\lambda_{bf}$ when the fluid-wall interaction is weak. It is shown by $r_{q}$ that the present calculated mass flow rate through the channel is smaller than the result calculated from the conventional hydrodynamic flow theory and this indicates that the formation of the physical adsorbed layer on the wall surface reduces the flow rate through the channel. However, when the fluid-wall interaction is weak, the present calculation is close to the conventional continuum flow calculation, and this indicates that the physical adsorbed layer is not so thick and it can flow well. Figure 2a shows that the value of $r_{q,s}$ is much smaller than that of $r_{q}$ and it is significantly lower than unity for $\lambda_{bf} \ge 0.03$. This suggests that when the fluid-wall interaction is weak, treating the physical adsorbed layer as a solid layer will result in an erroneous calculation of the flow rate through the channel and it will severely underestimate the flow rate for $\lambda_{bf} \ge 0.03$. Only for a very small $\lambda_{bf}$ (below 0.01) i.e. for a sufficiently large $h$, the physical adsorbed layer can be treated as a solid layer even when the fluid-wall interaction is weak. It is found from Fig. 2a that the portion of the total flow rate of the two adsorbed layers i.e. the value of $r_{b/h}$ is significantly increased with the increase of $\lambda_{bf}$ and this corresponds to the reduction of the value of $r_{q}$ and the increased deviation of $r_{q,s}$ from $r_{q}$. This manifests that with the increase of $\lambda_{bf}$ i.e. with the reduction of the continuum fluid film thickness $h$, the effect of the adsorbed layer on the mass flow rate through the channel is increased and the conventional hydrodynamic flow theory can not handle the adsorbed layer flow. For a considerable adsorbed layer flow, it is obvious that the adsorbed layer cannot be treated as a solid layer. These calculation results for the weak fluid-wall interaction should be qualitatively correct.

Figure 2b shows the calculated values of $r_{q}$, $r_{q,s}$ and $r_{b/h}$ for varying $\lambda_{bf}$ when the fluid-wall interaction is medium. For the same continuum fluid film thickness $h$, the value of $r_{q}$ in Fig. 2b is significantly smaller than that in Fig. 2a. This shows the considerably thicker physical adsorbed layer on the wall and the significantly stronger effect of the adsorbed layer for the medium fluid-wall interaction than for the weak fluid-wall interaction. For the medium fluid-wall interaction, conventional hydrodynamic flow theory much overestimates the flow rate through the channel for $\lambda_{bf} \ge 0.05$ owing to ignoring the adsorbed layer effect, which significantly reduces the flow rate as shown by the values of $r_{q}$(< 0.8) in Fig. 2b. The curves for $r_{q}$ and $r_{q,s}$ in Fig. 2b are much closer than those in Fig. 2a. This indicates the significantly increased solidification of the adsorbed layer for the medium fluid-wall interaction as compared for the weak fluid-wall interaction. Actually for $\lambda_{bf} \le 0.1$, the adsorbed layer can be treated as a solid layer for the medium fluid-wall interaction, as the values of $r_{q}$ and $r_{q,s}$ are close. However, for a low value of the continuum fluid film thickness $h$ which gives the value of $\lambda_{bf}$ as high as over 0.1, the physical adsorbed layer still can not be treated as a solid layer even for the medium fluid-wall interaction, as the value of $r_{q}$ is significantly greater than that of $r_{q,s}$; it suggests that for this case the adsorbed layer should still be considered as a flowing layer, otherwise the total mass flow rate through the channel will be pronouncedly underestimated. This corresponds to the considerable proportion of the flow rate of the adsorbed layer as shown by the values of $r_{b/h}$.

Figure 2c shows the significantly lower values of $r_{q}$ for the same value of the continuum fluid film thickness $h$ for the strong fluid-wall interaction than for the weak and medium fluid-wall interactions. This shows the strong effect of the adsorbed layer, which greatly reduces the flow rate through the channel, when the fluid-wall interaction is strong. Actually, Fig. 2c shows that the curves for $r_{q}$ and $r_{q,s}$ are quite close for the plotted $\lambda_{bf}$ values. Figure 2c obviously shows that the adsorbed layer can be treated as a solid layer for the strong fluid-wall interaction, when the continuum fluid film thickness $h$ is as high as to give the value of $\lambda_{bf}$ no more than 0.4.

The results in Fig. 2c qualitatively agree with the flow rates through the micro/nano channel calculated from the molecular dynamics simulation (MDS) by Liu and Li^9^ for the strong fluid-wall interaction ($\varepsilon_{fw} /KT = 10$) for wide channel heights (ranging from a few fluid molecule diameters to more than 250 fluid molecule diameters). Liu and Li^9^ suggested that there exists a strongly solidified layer on the channel wall for the strong fluid-wall interaction. By neglecting the effective immobile solidified layer thickness and using the effective channel height, they used the conventional continuum flow theory to re-calculate the flow rate through the channel (as like the calculated values of $r_{q,s}$ in the present study) and found that the calculation results are very close to the MDS results when the channel height is larger than 50 times of the fluid molecule diameter. Figure 2c shows that the values of $r_{q}$ and $r_{q,s}$ are particularly close for $\lambda_{bf} < 0.2$ (corresponding to the channel height larger than 7 times of the solidified layer thickness or 57 times of the fluid molecule diameter). This agreement actually proves the correctness of the present calculation for the strong fluid-wall interaction for $\lambda_{bf} < 0.2$. Liu and Li^9^ also showed that for the strong fluid-wall interaction, when the channel height is about over 175 times of the fluid molecule diameter, the flow rate calculated by MDS is nearly equal to that calculated from the conventional continuum flow theory. This corresponds to the present calculation result that the adsorbed layer effect is negligible and the flow rate through the channel can be calculated from the continuum flow theory when the channel height is large enough. Liu and Li^9^ showed that when the channel height is smaller than 12 times of the fluid molecule diameter, the flow rate through the channel calculated based on the solid layer assumption is a bit lower than that calculated by MDS especially for small channel heights. This follows the results shown in Fig. 2c that the value of $r_{q,s}$ is increasingly lower than the value of $r_{q}$ when $\lambda_{bf}$ is increased in the range $\lambda_{bf} > 0.3$ i.e. the channel height is reduced in the range of small values. Liu and Li^9^ ascribed this discrepancy to the calculation error occurring in MDS. However, the present study indicates that when calculating the flow rate through the channel for small channel heights i.e. high $\lambda_{bf}$ values, the flowing property of the adsorbed layer should still be considered even for the strong fluid-wall interaction.

## Conclusions

The multiscale analytical results are presented for the mass flow rate through a narrow slit pore when the effect of the physical adsorbed layer on the wall surface is considered. Intermediate between the adsorbed layers are the continuum fluid flow. The flow factor approach model for nanoscale flow handles the flow of the adsorbed layer, and the continuum model handles the continuum fluid flow. The explicit closed-form flow equations are respectively given for the flows of the two adsorbed layers and the flow of the continuum fluid; these equations all show the coupled effects between the adsorbed layer and the intermediate continuum fluid.

The calculation results show that the formation of the physical adsorbed layer on the wall surface reduces the flow rate through the channel. When the fluid-wall interaction is weak, this effect may be quite modest, the adsorbed layer flows well and it cannot be treated as a solid layer if the film thickness of the intermediate continuum fluid is not so high as to give $\lambda_{bf} \ge 0.03$. When the fluid-wall interaction is medium, the effect of the adsorbed layer is much stronger and it significantly reduces the flow rate through the channel when $\lambda_{bf} \ge 0.05$. For the intermediate continuum fluid film thickness as high as to give $\lambda_{bf} \le 0.1$, the adsorbed layer can be treated as a solid layer for the medium fluid-wall interaction; otherwise, it should still be treated as a flowing layer. When the fluid-wall interaction is strong, the strong adsorbed layer effect greatly reduces the flow rate through the channel for a practical narrow channel with the channel height on the scales of 10 nm or 100 nm; for this case, the adsorbed layer can be treated as a solid layer if the continuum fluid film thickness is not so low as to give the ratio $\lambda_{bf} \le 0.4$.

For a narrow channel flow where the effect of the adsorbed layer is considerable, there should be a method to incorporate the adsorbed layer effect. The adsorbed layer can be treated as a solid layer if the channel height is sufficiently large depending on the fluid-wall interaction, or it should be treated as a flowing layer because the flow of the adsorbed layer is comparable with that of the intermediate continuum fluid. For the latter, an efficient multiscale approach is required for estimating the adsorbed layer effect especially in engineering. The present study presents such an approach.
